# Nanomaterials targeting iron homeostasis: a promising strategy for cancer treatment

**DOI:** 10.3389/fbioe.2025.1511197

**Published:** 2025-03-12

**Authors:** Bin Li, Bing Zhang, Ziyue Cheng, Yantao Lou, Shuqiu Chen

**Affiliations:** ^1^ Institute of Urology, Zhong Da Hospital, Southeast University School of Medicine, Nanjing, China; ^2^ Department of Urology, Rushan Hospital of Traditional Chinese Medicine, Weihai, China; ^3^ Department of Urology, Yantai Yuhuangding Hospital, Qingdao University, Yantai, China

**Keywords:** iron metabolism, transferrin receptors, cancer therapy, iron chelators, nanocarriers

## Abstract

Iron is essential for vital cellular processes, including DNA synthesis, repair, and proliferation, necessitating enhanced iron uptake and intracellular accumulation. Tumor cells, in particular, exhibit a pronounced elevation in iron uptake to sustain their continuous proliferation, migration and invasion. This elevated iron acquisition is facilitated predominantly through the upregulation of transferrin receptors, which are closely associated with tumorigenesis and tumor progression. Incorporating transferrin into drug delivery systems has been shown to enhance cytotoxic effects in drug-sensitive cancer cells, offering a potential method to surpass the limitations of current cancer therapies. Intracellular iron predominantly exists as ferritin heavy chain (FTH), ferritin light chain (FTL), and labile iron pool (LIP). The innovation of nanocarriers incorporating iron chelating agents has attracted considerable interest. Iron chelators such as Deferoxamine (DFO), Deferasirox (DFX), and Dp44mT have demonstrated significant promise in cancer treatment by inducing iron deficiency within tumor cells. This review explores recent advancements in nanotechnology aimed at targeting iron metabolism in cancer cells and discusses their potential applications in cancer treatment strategies.

## 1 Introduction

Iron plays an essential role in regulating various activities within human life, including hemoglobin synthesis, energy metabolism, and DNA synthesis (Morales and Xue, 2021). Cells maintain iron content within a specific range to support normal cellular functions. Abnormal iron levels can significantly impact cells. Low iron levels can disrupt various biological processes, including enzymes activity, oxygen transport, heme synthesis, and detoxification processes (Biz and Mahadevan, 2021; Carpenter and Payne, 2014; Dutt et al., 2022; Ruiz et al., 2021). Conversely, high iron concentrations can pose a cancer risk due to its prooxidant activity, which can cause oxidative DNA damage. Iron predominantly exists in a protein-bound form, including in heme compounds like hemoglobin, ferritin (FT), hemosiderin, and myoglobin in erythrocytes (Salnikow, 2021). Only a small fraction of unbound iron exists in the cytoplasm, referred to as the labile iron pool (LIP) (Boccio et al., 2003; Vogt et al., 2021). Given iron’s crucial role in life activities, sophisticated feedback mechanisms for iron homeostasis are present in the body, including iron absorption by organs, systemic transportation, and cellular uptake and storage.

**FIGURE 1:**
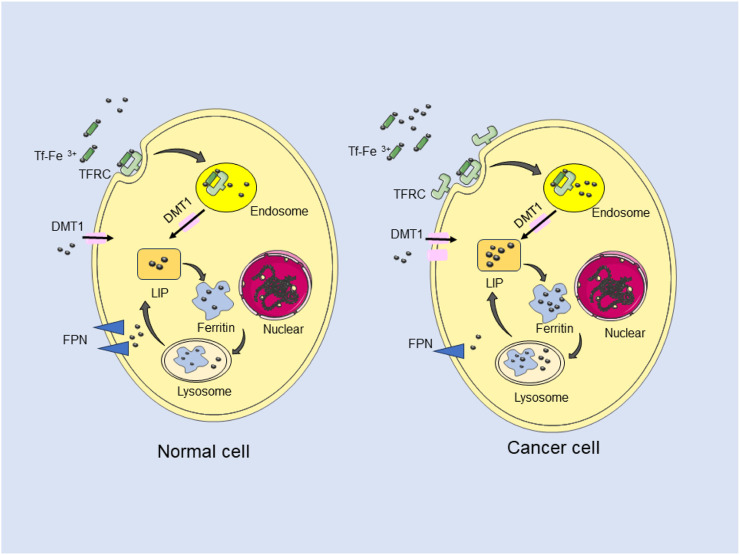
Iron metabolism in cancer cells. Iron was transferred inside cancer cells via internalization by TFRC and DMT1. Iron was exported out with FPN. Intracellular iron was stored as Ferritin and LIP. Cancer cells require large amount of iron than normal cells.

Iron metabolism plays a dual role in cancer biology, both promoting the proliferation and metastasis of tumor cells and inducing ferroptosis to inhibit their malignant traits (Wang et al., 2016; Rochette et al., 2022). Iron is critical for cell proliferation, and tumor cells, compared to normal cells, require increased iron uptake to maintain their growth, migration and invasion. Epidemiological studies have established a positive correlation between dietary iron intake and systemic iron levels with the incidence of various cancers, including colorectal, pancreatic, lung, and bladder cancers (Huang et al., 2023; Khan and Sharma, 2023; Wang et al., 2022; Qin et al., 2024). Divalent metal transport 1 (DMT1), a cellular iron transporter, facilitates iron uptake by small intestinal epithelial cells and mediates the transfer of iron from endosomes to the cytoplasm (Yanatori and Kishi, 2019). Targeted knockout of DMT1 inhibits iron uptake by colon epithelial cells, disrupts the iron-regulated signaling pathway mediated by CDK1, JAK1 and STAT3, and consequently suppresses tumor cell proliferation in colon cancer mouse models, thereby reducing tumor burden (Cheli et al., 2018). conversely, high concentrations of iron can exert cytotoxic effects, leading to ferroptosis. Various ferroptotic inducers, including piperazine and pharmaceutical agents like sorafenib, statins, and sulfasalazine, along with cytokines such as IFN-γ and TGF-β1, have been demonstrated to induce ferroptosis in tumor cells, thus hindering tumor proliferation (Yang et al., 2014). Tumor cells, however, have developed strategies to evade ferroptosis, such as preventing membrane damage and reducing intracellular peroxide accumulation through the uptake of extracellular cysteine, thereby circumventing ferroptosis. These insights highlight the complex role of iron metabolism in cancer development and therapy (Figure 1).

Ferroptosis is an emerging mode of programmed cell death, distinctively characterized by iron-dependent lipid peroxidation and the substantial accumulation of reactive oxygen species (ROS) (Mou et al., 2019; Tang and Kroemer, 2020). Unlike necrosis and apoptosis, ferroptosis exhibits unique morphological and functional characteristics. It lacks typical necrotic features such as cytoplasmic and organelle swelling and membrane rupture, as well as apoptotic features like cell shrinkage, chromatin condensation, and apoptotic body formation (Ai et al., 2024). Instead, ferroptosis primarily manifests as mitochondrial shrinkage, increased membrane density, reduced or absent mitochondrial cristae, rupture of the extracorporeal membrane, depletion of the reducing agent glutathione (GSH), and increased ROS levels (Mou et al., 2019). Research on drug development targeting ferroptosis is gaining traction as a promising anticancer therapeutic strategy (Yang et al., 2014; Greenshields et al., 2017). A variety of approaches, including the application of clinical drugs, experimental small molecule compounds, ferroptosis-related genes, and nanomaterials, have been explored to induce ferroptosis in tumor cells (Liang et al., 2023). These advancements underscore the potential of targeting ferroptosis as an innovative approach in cancer treatment.

Nanoparticles are employed in the delivery and control of therapeutic agents due to their outstanding characteristics. These include targeted delivery, which ensures precise delivery to specific sites, and controlled release, which allows for sustained therapeutic effects. Nanoparticles also exhibit high biocompatibility and low toxicity, thereby minimizing adverse effects on healthy tissues. Additionally, their ability to degrade within a clinically acceptable timeframe mitigates the risk of long-term accumulation in the body. These properties position nanoparticles as a promising platform in the development of advanced therapeutic strategies (Zaimy et al., 2017; Baetke et al., 2015). The utilization of nanoparticles as carriers for chemical or biological materials presents a significant opportunity to enhance the efficacy of existing ferroptosis inducers and facilitate the development of novel inducers for cancer treatment. By leveraging the targeted delivery and controlled release capabilities of nanoparticles, it is possible to improve the therapeutic index of ferroptosis inducers, thereby maximizing their anticancer effects while minimizing off-target toxicity. Furthermore, the adaptability of nanoparticles allows for the incorporation of a wide range of active agents, paving the way for innovative approaches in the induction of ferroptosis and the advancement of cancer therapies (Alavi and Hamidi, 2019; Ashrafizadeh et al., 2019).

The integration of innovative approaches in cancer therapy has driven considerable interest in exploring the unique characteristics of tumor cell biology, particularly in relation to iron metabolism. Tumors exhibit an elevated iron uptake compared to normal cells, a trait that not only supports their proliferation but also positions iron metabolism as a promising therapeutic target (Morales and Xue, 2021). Ferroptosis, a form of programmed cell death characterized by iron-dependent lipid peroxidation, represents a key area of interest for its potential to selectively eliminate cancer cells. Current strategies to induce ferroptosis focus on either depriving tumor of iron or augmenting iron levels to induce cytotoxicity. While both methods hold promise, further research is needed to determine their relative efficacy in clinical settings. In this context, nanoparticle technology emerges as a powerful tool, offering enhanced delivery and control of ferroptosis inducers through its characteristics of targeted and controlled release, biocompatibility, and low toxicity. By coupling nanoparticles with chemical or biological agents, the effectiveness of existing ferroptosis inducers may be significantly improved (Zaffaroni and Beretta, 2021). Moreover, manipulating proteins involved in the ferroptosis pathway can modulate intracellular iron levels and disrupt the cellular REDOX balance, triggering ferroptosis in tumor cells. The potential to combine these novel tactics with traditional anti-tumor therapies could yield synergistic effects, enhancing overall treatment efficacy and potentially overcoming resistance associated with conventional methods. This review explores the multifaceted approaches to targeting iron metabolism and ferroptosis in cancer therapy, highlighting the potential of nanoparticles and pathway-specific interventions to revolutionize treatment paradigms.

## 2 Iron Homeostasis in Cancer: CSCs, EMT, and Chemoresistance

### 2.1 Iron regulation of cells

Iron metabolism in the human body encompasses several processes, including absorption, transport, utilization, loss, circulation, regulation, and storage. Maintaining Iron homeostasis is crucial to ensure adequate iron for essential biological functions while preventing toxicity from excess iron (Boccio et al., 2003). The oxidation state of iron significantly influences its absorption in the gastrointestinal tract, requiring it to be in the ferrous form (Fe2+) or bound to transporters for absorption. Dietary ferric iron (Fe3+) must be reduced to ferrous iron before uptake through divalent metal transporter 1 (DMT1) into intestinal epithelial cells. In contrast, If haem iron can be absorbed directly absorbed via haem carrier protein 1(HCP1) (Cheli et al., 2018). Upon absorption into intestinal epithelial cells, a portion of iron is stored as ferritin, while the remaining iron is transported into the circulation through ferroportin, located on the basolateral membrane. During this process, iron is reoxidized to its ferric form with the assistance of ferroportin auxiliary proteins, enabling it to bind to transferrin in the bloodstream. This binding facilitates the transport of iron to target organs for its biological roles. Hepcidin and transferrin are pivotal regulators of iron transport from cells to systemic circulation. Hepcidin, synthesized by the liver, is upregulated in response to increased iron levels, such as elevated iron stores and serum iron levels, as well as during infection and chronic inflammation, thereby playing a crucial role in maintaining iron homeostasis. Elevated hepcidin levels interact directly with membrane-bound iron transport protein (FPN), facilitating internalization and subsequent degradation. This process ultimately inhibits the efflux of iron into the bloodstream, thereby reducing circulating iron levels and contributing to iron homeostasis regulation (Nemeth and Ganz, 2021). Iron regulatory proteins (IRPs) bind to iron responsive elements (IREs), which are sequences found in mRNAs that encode iron-related genes. This binding controls the expression of these genes, including hypoxia inducible factor 2α (HIF2α) and transferrin receptor 1 (TfR1). Through this IRP/IRE posttranscriptional regulatory system, the cellular iron storage and homeostasis are effectively managed, ensuring appropriate iron levels for various cellular functions (Khan and Sharma, 2023; Sanchez et al., 2006). Iron is transported through the bloodstream by binding to transferrin, which delivers it to target organs. At these sites, iron binds to transferrin receptors on the cell surface and is internalized via clathrin-dependent endocytosis. Iron was delivered to different parts of the cell including the mitochondria by proteins poly (rC)-binding proteins 1 and 2 (PCBP1 and PCBP2) (Frey et al., 2014; Ryu et al., 2017). Mitoferrin 1 imports iron from intermembrane space to mitochondrial matrix for the synthesis of Fe S clusters. Once inside the cell, iron dissociates from transferrin and is reduced back to its ferrous form (Fe2+). The ferrous iron is then transported into the cytoplasm by DMT1 and can either be utilized in the mitochondria for various biochemical processes or stored as ferritin for future use (Barra et al., 2024; Gao et al., 2019).

### 2.2 Iron homeostasis in cancer cells

High concentrations of iron are associated with carcinogenesis, a finding supported by epidemiological studies. In cancer cells, iron homeostasis is disrupted, resulting in elevated intracellular iron levels. Cancer cells, characterized by their rapid proliferation and heightened energy metabolism, exhibit a substantially increased demand for iron (Marques et al., 2014). To accommodate this demand, these cells predominantly store iron in the form of ferritin, consisting of both ferritin light chain (FTL) and heavy chain (FTH). Additionally, cancer cells exhibit a significant increase in the labile iron pool (LIP), which further contributes to their iron dependency. Iron uptake from the microenvironment is predominantly facilitated by the overexpression of TfR1 on the cell surface. This increased expression of TfR1 is crucial in enhancing cancer cell proliferation and invasion, making it an important target for the development of therapeutic strategies in cancer treatment. By focusing on TfR1, researchers aim to disrupt the iron acquisition pathway essential for tumor growth, presenting a promising avenue for effective cancer therapies. The Increased expression of import proteins, such as DMT1 and TFR1, has been demonstrated in colorectal cancer, leading to elevated intracellular iron. The STEAP family of proteins, which are involved in iron uptake and reduction within endosome, are also high expressed in various tumors, including glioma, prostate, pancreatic, and breast cancer (Rocha, 2021; Gomes et al., 2012; Chen et al., 2021). This elevated expression highlights the importance of the STEAP proteins in tumor iron metabolism and their potential as targets for therapeutic interventions in these cancers. Tumor cells were characterised of rapid self-renewal, therefore higher levels of nutrition were required compared to normal cells. Mitochondria was important organelles in process of energy synthesis. Iron plays an important role in mitochondrial enzyme synthesis. Excess iron was delivered to different parts of the cell including the mitochondria by PCBP1 and PCBP2. Mitoferrin 1 imports iron from intermembrane space to mitochondrial matrix for high concentration of Fe S clusters (Chung et al., 2014). Excess iron in mitochondria is also stored in ferritin (Levi and Arosio, 2004). Iron efflux is controlled by FPN, which is regulated by hepcidin. In many cancer cells, the low expression of FPN induces an increase level of intracellular iron (Lehmann et al., 2023). This is further compounded by the high expression of Hepcidin, which suppresses FPN and reduces iron export. Contrary to this, most cancer types exhibit high expression of iron import genes and low expression of iron export and storage genes, leading to an accumulation of iron within the cells. This dysregulation in iron homeostasis contributes to cancer cell proliferation and survival (Roth et al., 2019).

### 2.3 Iron homeostasis in relation to CSCs, EMT, and chemoresistance

Iron is essential for DNA synthesis, repair, and cellular proliferation, which results in increased in iron uptake and elevated intracellular iron concentrations. Intracellular iron consists of components like ferritin heavy chain (FTH), ferritin light chain (FTL), and labile iron pool (LIP). The transferrin receptor, a membrane protein that binds transferrin, facilitates this high iron concentration typically observed in cancer cells (Jia et al., 2024; Rosager et al., 2017). TFRC is highly expressed in MXR-resistant cells, whereas its concentration is low in drug-resistant patients (Yu et al., 2024). To explore the relationship between iron metabolism and chemoresistance, studies on doxorubicin-resistant and cisplatin-resistant MCF-7 cell lines have shown elevated expression of TFRC1 and iron-regulating genes. Iron chelation leads to decreased levels of cycline A, B and D, resulting in G1/S phase arrest and inducing cell apoptosis (Kulp et al., 1996). Depletion iron disrupts the iron metabolism in cancer cells, which can help reduce drug resistance. The LIP is crucial for the proliferation of cancer cells, and iron chelators can effectively bind to the free iron within this pool, thereby inhibiting cell growth and proliferation. This strategy not only hampers the metabolic processes essential for cancer cell survival but also enhances the effectiveness of chemotherapeutic agents by overcoming resistance mechanisms. Iron chelator (DFO and DFX) enhance chemotherapy sensitivity (Wang et al., 2019; Liu et al., 2022). While High intracellular iron concentrations support the growth and proliferation of malignant cells, iron chelating agents inhibit tumor growth by depleting intracellular iron. Previous studies have demonstrated the anticancer effects of the iron chelator deferoxamine (DFO), which increases the chemotherapy sensitivity of ovarian cancer by inducing apoptosis in tumor cells. These findings suggest that depleting iron within cells can mitigate chemotherapy resistance, highlighting the potential role of iron chelation in cancer treatment strategies.

The stemness of cancer stem cells (CSCs) is associated with elevated levels of ferritin, both FTH and FTL, capable of storing more than 4,000 iron atoms. High ferritin levels are particularly linked to breast CSCs, whereas knockdown of FTH disrupts the expression of the cytokine oncostatin M and impairs stemness. FPN regulates the export of excess intracellular iron, a process modulated by hepcidin (HAMP). In breast cancer, high expression of HAMP and an increased LIP are correlated with poor prognosis and an invasive phenotype, alongside downregulated FPN expression. Overall, CSCs maintain high iron concentrations by enhancing iron uptake, reducing iron export, and stabilizing LIP homeostasis, contributing to their malignancy and resistance characteristics (Arruda et al., 2020).

Post-transcriptional regulation of iron in cancer stem cells (CSCs) is primarily mediated by iron regulatory proteins 1 and 2 (IRP1 and IRP2), which are RNA-binding proteins that play a critical role in iron metabolism. In non-CSCs, the Lip is enhanced by the proliferation-associated gene c-Myc. IRP1 bind to IREs, modulating the expression of genes related to iron metabolism and CSCs characteristics. CSCs are characterized by increased iron influx and decreased iron efflux, resulting in elevated intracellular iron. High levels of heavy-chain ferritin (H-ferritin) and intracellular iron is closely associated with CSC features in cancer. Iron supplementation significantly contributes to the maintenance of stemness and promotes chemoresistance in CSCs. The elevated iron levels in CSCs are critical, as evidenced by the upregulation of TFRC and DMT1, which enhance iron uptake and intracellular levels, thereby facilitating growth and maintaining CSC stemness (Brown et al., 2019; Lee and Roh, 2023).

## 3 Iron-Targeting Nanotherapeutics for Enhanced Cancer Treatment

### 3.1 Transferrin-conjugated nanocarriers serve as precision drug delivery systems for enhanced cancer therapy

Nanomaterial-based therapeutic compound delivery system has been explored to enhance therapeutic specificity and minimize systemic toxicity through the design of targeted therapy strategies. TFRC, which are iron-binding proteins located on cell membrane, play a crucial role in transporting iron necessary for cell proliferation. There receptors are expressed at low levels in normal cells but are significantly upregulated in malignant cells, including those from prostate, breast, pancreatic, leukemia, colon, and lung cancers (Currie et al., 2023; Yang et al., 2022; Liu et al., 2023; Zhang et al., 2024). In cancer cells, increased iron uptake via transferrin receptors has been observed, with accumulating evidence indicating that TFR1 is implicated in tumorigenesis and progression. The association between TFR1 and cancer underscores its potential as a drug target for cancer therapy (Candelaria et al., 2021). There are two subtypes of TFR, TFR1 and TFR2. TFR1 is generally expressed on the surface of most cells, whereas TFR2 is predominantly expressed in liver cells. These receptors are membrane glycoproteins that facilitate iron uptake via transferrin-bound iron (Fe III). The Tf-TFR1 complex is internalized through endocytosis, after which Fe (III) dissociates from TF. Given the high expression of TFRC in tumor cells, specific ligands targeting TFRC have been utilized for surface modification of nanomaterials, thereby to enhance selectivity and accumulation, thereby improving therapeutic efficacy.

The conjugation of transferrin with anti-transferrin receptor peptides has been investigated to enable selective drug delivery for tumor therapy (Nogueira-Librelotto et al., 2017). This drug delivery system enhances cytotoxicity in both drug-sensitive and drug-resistant cells, thus addressing limitations associated with current cancer management strategies. Overall, targeted transferrin therapy, leveraging the iron metabolism of cancer cells, has significantly advanced nanoparticle research.

### 3.2 Iron chelation targeting strategies affecting cancer treatment

**FIGURE 2:**
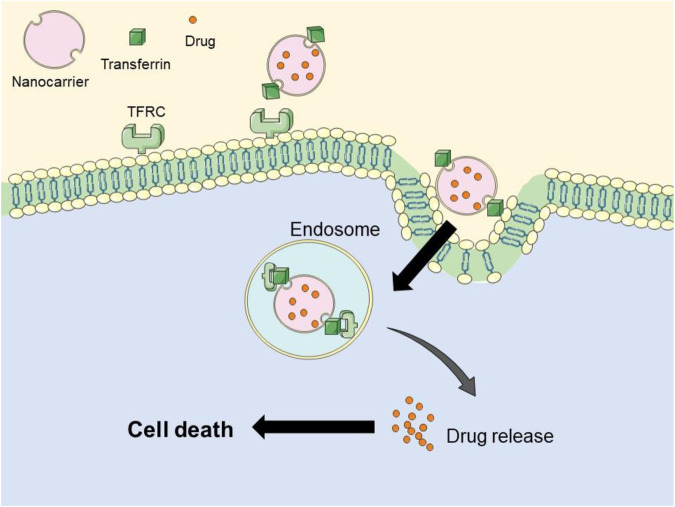
Pattern diagram of transferrin-targeted nanomaterial. Surface of nanomaterial was modified with transferrin, linked with transferrin receptor of cancer cells, resulting in internalization by endocytosis.

Nanocarriers incorporating iron chelating agents have been extensively investigated for their potential in cancer treatment. Chelators such as DFO, deferasirox (DFX), and Dp44mT have shown promise in inducing iron deficiency within tumor cells, thereby inhibiting their proliferation (Ibrahim and O'Sullivan, 2020). However, the therapeutic efficacy of these iron chelating agents is limited by their short half-lives. DFO exhibits a half-life of approximately 20 min, which results in minimal impact on the iron levels within tumor cells. Considering the non-specificity of iron chelating agents in cancer therapy, the incorporation of nanomaterials offers a promising strategy to extend the drug cycle duration of these agents, leading to more effective cancer treatment (Ibrahim and O'Sullivan, 2020). Nanoparticles were engineered to prolong circulation time, enhance clearance, improve biosafety, and increase cellular permeability. Given the role of iron in promoting cancer progression in pancreatic tumors, a nanomaterial based on iron chelation has been developed. Liposomal drug delivery systems have been explored for the encapsulation of YC-1, a known inhibitor that targets DFO, transferrin (TF), and hypoxia-inducible factor 1α (HIF-1α). Transferrin modified nanomaterials enhance the expression specificity of membrane protein TFRC (Figure 2). Additionally, the iron chelating agent DFO acts synergistically with YC-1 to treat cancer. Pancreatic cancer CSCs exhibit elevated iron levels, and this novel, effective combined delivery of an iron chelator and YC-1 significantly enhances the efficacy of chemotherapy for pancreatic cancer (Lang et al., 2019).

Iron supply contributes to the growth and proliferation of stem cells, and iron derivatives specifically target stem cells can induce cancer cell death. Nanoparticulate deferoxamine (Nano DFO) has been formulated into polyethylene glycol lipid nanocapsules (LNCs) to enhance therapeutic efficacy. LNCs offer improved bioavailability, biosafety and anticancer effects. Compared to conventional iron chelating agents, the nanostructured formulation of DFO exhibits superior anti-tumor activity both *in vivo* and *in vitro*. Despite its effectiveness, the DFO family is still limited by challenges such as rapid clearance, poor cellular permeability, and cytotoxicity. To address these limitations, researchers have developed novel DFO derivatives that target CSCs by conjugating DFO with caffeine, thus improving permeability and targeting efficiency (Li et al., 2019). The newly developed compound demonstrates excellent cellular permeability and safety in depleting intracellular iron. The new derivative, DFCAF, surpasses DFO in effectively targeting cellular iron concentrations. DFCAF inhibits the growth and invasiveness of CSCs by suppressing the expression of the TGF-β signaling pathway.

### 3.3 Induction of ferroptosis in cancer cells via nanoparticle strategies

Ferroptosis is a distinctive form of cell death reliant on the presence of iron and reactive oxygen species (ROS) (Park and Chung, 2019). In recent years, nanomedicine has emerged as a promising strategy for the effective treatment of various cancers using engineered nanomaterial-based therapeutic reagents. Increasing research has highlighted the significant relationship between ferroptosis and nanomedicine. Due to their nanoscale size, engineered nanomaterials can passively target tumor tissues through enhanced permeability and retention (EPR) effects, facilitating cancer-specific therapy (Alphandery, 2022). In addition, iron-containing nanomaterials can enhance ROS accumulation following cellular uptake, ultimately resulting in cell death and achieving therapeutic effects. However, the ferroptosis inducer Solanine A presents challenges due to its poor water solubility and high toxicity in animal studies. Utilizing amphiphilic biodegradable ph-sensitive nanocarrier can mitigate the adverse pharmacological profiles associated with certain therapeutic agents (Hassannia et al., 2018). For instance, In a leukemia cell xenotransplantation model, the antitumor activity of the erastin analogue IKE was enhanced when delivered via polyethylene glycol-polylactic-coglycolic acid nanoparticles (Zhang et al., 2019). Additionally, in xenograft models, ultra-small silica nanoparticles have been shown to induce ferroptosis by increasing intracellular iron transfer and accumulation, thereby inhibiting tumor growth (Kim et al., 2016). Li et al. (2022) devised and developed engineered exosome that were endogenously modified with brain tumor-targeting peptides and combined with magnetic nanoparticles through antibody conjugation. This platform enabled the loading of siGPX4 and Brequinar (BQR), an DHODH inhibitor, on exosomal surfaces and mesoporous silicon, respectively. Furthermore, a mouse magnetic helmet constructed using 3D printing technology facilitated the effective induction of ferroptosis for treating brain gliomas. nevertheless, the long-term effects of nanoparticle applications on human health require careful evaluation.

## 4 Conclusion

The advancements in nanotechnology have significantly enhanced the potential for effective cancer treatment strategies. Engineered nanomaterials, due to their nanoscale size and unique properties, enable targeted delivery and enhanced retention in tumor tissues, providing a promising platform for selective cancer treatment. The integration of iron-containing nanomaterials, amphiphilic biodegradable pH-sensitive carriers, and sophisticated delivery systems such as modified exosomes have demonstrated increased therapeutic efficacy in various preclinical models by capitalizing on the ferroptosis pathway. Despite these advancements, challenges remain, particularly concerning the pharmacological profiles and potential long-term effects on human health. Further research is essential to optimize these systems and ensure their safety and effectiveness in clinical settings. The continued exploration of nanoparticle-based strategies holds great potential for transforming cancer treatment modalities, providing new avenues for overcoming drug resistance and enhancing patient outcomes.
